# Effect of Different In_2_O_3_(111) Surface Terminations on CO_2_ Adsorption

**DOI:** 10.1021/acsami.3c07166

**Published:** 2023-09-13

**Authors:** Sabrina M. Gericke, Minttu M. Kauppinen, Margareta Wagner, Michele Riva, Giada Franceschi, Alvaro Posada-Borbón, Lisa Rämisch, Sebastian Pfaff, Erik Rheinfrank, Alexander M. Imre, Alexei B. Preobrajenski, Stephan Appelfeller, Sara Blomberg, Lindsay R. Merte, Johan Zetterberg, Ulrike Diebold, Henrik Grönbeck, Edvin Lundgren

**Affiliations:** ‡ Division of Combustion Physics, Lund University, 22100 Lund, Sweden; § Department of Physics and Competence Centre for Catalysis, Chalmers University of Technology, 41296 Göteborg, Sweden; ¶ Institute of Applied Physics, Technische Universität Wien, 1040 Vienna, Austria; ∇ MAX IV Laboratory, Lund University, 22100 Lund, Sweden; ∥ Department of Chemical Engineering, Lund University, 22100 Lund, Sweden; ⊥ Department of Materials Science and Applied Mathematics, Malmö University, 20506 Malmö, Sweden; # Division of Synchrotron Radiation Research, Lund University, 22100 Lund, Sweden

**Keywords:** X-ray photoelectron spectroscopy, core-level shifts, heterogeneous catalysis, density functional theory, indium oxide, CO_2_ adsorption, methanol synthesis

## Abstract

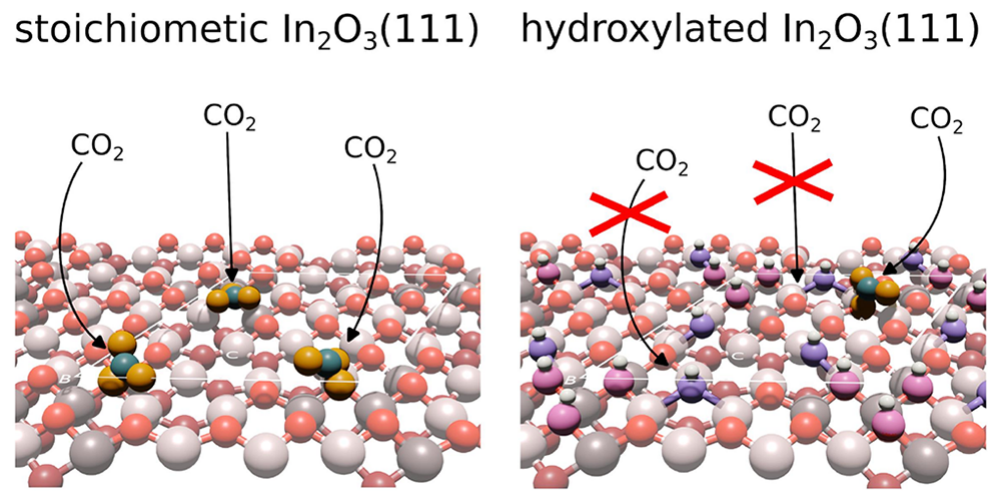

In_2_O_3_-based catalysts have shown high activity and selectivity for CO_2_ hydrogenation to methanol; however, the origin of the high performance of In_2_O_3_ is still unclear. To elucidate the initial steps of CO_2_ hydrogenation over In_2_O_3_, we have combined X-ray photoelectron spectroscopy and density functional theory calculations to study the adsorption of CO_2_ on the In_2_O_3_(111) crystalline surface with different terminations, namely, the stoichiometric, reduced, and hydroxylated surface. The combined approach confirms that the reduction of the surface results in the formation of In adatoms and that water dissociates on the surface at room temperature. A comparison of the experimental spectra and the computed core-level shifts (using methanol and formic acid as benchmark molecules) suggests that CO_2_ adsorbs as a carbonate on all three surface terminations. We find that the adsorption of CO_2_ is hindered by hydroxyl groups on the hydroxylated surface.

## Introduction

The emission of greenhouse gases to the atmosphere has been identified as the origin of climate change.^1^ CO_2_ has been recognized as one of the main contributors to the greenhouse effect. One suggestion to mitigate its environmental impact is to capture CO_2_ from the atmosphere, which introduces the challenge to contain the captured CO_2_.^2^ An appealing solution to this issue is the catalytic conversion of CO_2_ to a more valuable fuel or platform chemical.^3^ The hydrogenation of CO_2_ using renewable H_2_ from water splitting is one promising route for the catalytical conversion of CO_2_ to useful oxygenates such as methanol (CH_3_OH), which is a key building block in the chemical industry and a renewable fuel.^4^ Methanol can be synthesized from CO_2_ hydrogenation by thermal catalysis,^5^ electrocatalysis^6,7^ and photocatalysis.^8^ In thermal catalysis, Cu–ZnO–Al_2_O_3_ catalysts are commonly used for methanol synthesis. These Cu–ZnO–Al_2_O_3_ catalysts suffer, however, from deactivation due to thermally induced sintering,^9^ agglomeration of ZnO species, and oxidation of metallic Cu.^10^

In_2_O_3_-based catalysts have been suggested as an alternative to Cu–ZnO catalysts. Recent investigations of ZrO_2_-supported In_2_O_3_ catalysts revealed high stability for CO_2_ hydrogenation under industrial relevant conditions (temperatures of 473–573 K and pressures of 1.0–5.0 MPa), as well as high activity and 100% selectivity for methanol synthesis.^11^ The origin of the high performance of ZrO_2_-supported In_2_O_3_ and the nature of the active sites of the catalysts have been the subjects of intense investigations. Martin et al. proposed^11^ that the high performance originates from the high concentration of oxygen vacancies in In_2_O_3_. However, these surface oxygen vacancies do not exist on stoichiometric In_2_O_3_(111) under ultrahigh-vacuum (UHV) conditions and could not be created by thermal reduction.^12^ Additionally, kinetic modeling based on density functional theory (DFT) suggests that oxygen vacancies are not crucial for the reaction but instead that a surface structure that allows for changes in the oxidation state of the In cations is needed.^13^ Furthermore, the quantification of oxygen vacancies is based on the appearance of an additional peak at higher binding energies in the O 1s X-ray photoelectron spectroscopy (XPS) spectra. However, previous and current calculations on the O 1s core-level shifts (CLSs) of In_2_O_3_ surfaces show that those peaks should be assigned to hydroxyl groups rather than oxygen vacancies.^14,15^

Different atomic-scale reaction pathways have been suggested for the CO_2_-hydrogenation reactions. One is known as the reverse water–gas shift (RWGS) reaction, which involves the dissociation of CO_2_ and hydrogenation to methanol via a formyl (HCO) intermediate, whereas a more direct reaction pathway via the formation of formate (HCOO^–^) has also been discussed in the literature.^4^ The high selectivity of In_2_O_3_-based catalysts has been attributed to the suppression of the RWGS reaction,^16^ while the origin of RWGS suppression remains a subject of debate. Additionally, the effect of water on CO_2_ hydrogenation has been discussed, and the inhibition of CO_2_ hydrogenation by water has been reported,^11^ although the underlying reason behind this effect remains unknown.

Fundamental investigations of a well-defined single-crystalline In_2_O_3_ surface in a controlled environment could advance the understanding of active sites and the effect of water on CO_2_ hydrogenation. The In_2_O_3_(111) surface is the thermodynamically most stable surface of In_2_O_3_ and has, moreover, been suggested to be active for CO_2_ hydrogenation,^17^ making it of interest for fundamental and detailed investigations. In this paper, we apply combined experimental synchrotron-based XPS measurements and computational DFT calculations of well-defined In_2_O_3_(111) surfaces prepared under pristine (i.e., UHV) conditions. Different surface terminations of In_2_O_3_(111) were investigated,^12,18,19^ namely, the stoichiometric, reduced, and hydroxylated surfaces. In adatoms are identified via the In 3d core level on the reduced surface, and OH groups are identified via the O 1s core level on the hydroxylated surface. The XPS fingerprints of these different surface terminations will facilitate the interpretation of future experiments performed at higher pressures. This work focuses on the adsorption of CO_2_ on the stoichiometric, reduced, and hydroxylated surfaces under UHV conditions. We demonstrate that CO_2_ forms carbonates with lattice O atoms on all three surface terminations, but the presence of OH groups limits CO_2_ adsorption. Additionally, we investigated the adsorption of methanol and formic acid on the stoichiometric surface.

## Experimental and Computational Methods

The In_2_O_3_(111) films of 200 nm thickness were grown on yttria-stabilized zirconia by pulsed-laser deposition in Vienna, as described in the literature.^18^ The films are single-crystalline and exhibit atomically flat surfaces that can be prepared to exhibit different terminations following previously reported UHV treatments.^12,19^ The stoichiometric surface was prepared by gentle sputtering and subsequent annealing to 800 K in 2 × 10^–6^ mbar of O_2_ for 20 min and cooling in O_2_ to prevent adatom formation. The reduced In_2_O_3_(111) surface was obtained by annealing the stoichiometric In_2_O_3_(111) in UHV at 720 K for 30 min. The hydroxylated surface was prepared by exposing the stoichiometric In_2_O_3_(111) to 1 langmuir (1.33 × 10^–6^ mbar s) of H_2_O at room temperature. Prior to the adsorption experiments with methanol and formic acid, the liquids were cleaned by three freeze–pump–thaw cycles. The stoichiometric surface was flashed to 475 K to desorb any OH groups from the surface. Subsequently, the sample was cooled to room temperature. Once the surface had reached room temperature, 1 langmuir of methanol or formic acid was dosed through a leak valve with a pressure of 5 × 10^–9^ mbar.

The XPS measurements were performed at the Surface Materials Science branch of the FlexPES beamline at the MAX IV synchrotron.^20^ This beamline is dedicated to high-resolution XPS and soft-wavelength X-ray absorption measurements. The endstation is equipped with a Scienta DA-30 L analyzer and a preparation chamber with a low-energy electron diffraction (LEED) setup. We measured high-resolution XPS spectra of In 3d_5/2_ and O 1s with an excitation energy of 600 eV and C 1s at an energy of 400 eV to ensure high surface sensitivity. All In 3d_5/2_ and O 1s spectra were recorded with a pass energy of 20 eV and all C 1s with a pass energy of 50 eV. All spectra were recorded with an energy step size of 50 meV, and the binding energy was calibrated on the valence-band maximum (VBM) by setting it to 3.3 eV to compensate for band-bending effects.^21^

We observed minor potassium contamination on the sample, which accumulated on the surface when the sample was annealed. The contamination could be reduced by sputtering but not entirely removed because it returned with annealing. The amount of potassium on the surface was estimated from the C 1s and K 2p XPS spectra. Based on the peak area of the spectra and the photoionization cross section, the potassium coverage is approximately 8% of the saturation methanol coverage, which corresponds to three C atoms per unit cell. Thus, the K contamination amounts to approximately 0.06 K atoms per unit cell or ≈0.05 at./nm^2^. No other contaminants could be detected within the resolution limit of XPS. The ordering of the surface was ascertained by the presence of sharp LEED spots (Figure S1).

The fitting of the core-level spectra was performed using the *CasaXPS* software package, version 2.3.24.^22^ A Shirley background was applied to the In 3d_5/2_ spectra, and a linear background to the O 1s and C 1s spectra. The peak shapes that were used for the fitting are the sum of a Gaussian and Lorentzian “SGL(p)”, and an asymmetric Lorentzian line shape with tail damping “LA(α,β,m)”. Details on the fit functions can be found in the *CasaXPS* handbook.^22^

The Vienna ab initio simulation package (*VASP*, version 5.4.4)^23−26^ was used to perform DFT calculations with the Perdew–Burke–Ernzerhof (PBE)^27,28^ and Heyd–Scuseria–Ernzerhof (HSE06)^29−31^ functionals. The PBE functional was employed for all structure relaxations and O 1s and In 3d CLSs, whereas HSE06 was used in the CO_2_ adsorption calculations and the C 1s core-level spectra [see the Supporting Information (SI) for details]. The projector-augmented-wave (PAW) method was used to describe the interaction between the core and valence electrons^32^ together with a plane-wave basis set with a 500 eV cutoff energy to expand the Kohn–Sham orbitals. The valence was chosen to be 1s^1^, 2s^2^2p^2^, 2s^2^2p^4^, and 4d^10^5s^2^5p^1^ for H, C, and In. The optimized bixbyite bulk structure for In_2_O_3_ was obtained from our earlier work.^14^ The In_2_O_3_(111) surface was modeled with a 1 × 1 surface cell of a thickness of five trilayers (for surface termination studies and O 1s/In 3d CLSs) and three trilayers (saturation coverage calculations and all hybrid calculations), with two or one bottom layer fixed at the optimized bulk positions, respectively. A 3 × 3 × 1 Monkhorst–Pack mesh was used to sample the Brillouin zone for the PBE calculations, whereas hybrid calculations were performed using the Γ-point approximation. The CLS calculations included both initial and final state effects.^33−35^ The O 1s and In 3d shifts were computed as the difference in the energy of the system with a core hole on the atom of interest and the energy of the system with a core hole in a reference atom in the center of the slab representing bulk In_2_O_3_. The C 1s shifts were computed as the difference in the energy of the system with a core hole on the atom of interest and the energy of the system with a core hole in the C atom of a methoxy (OCH_3_) group placed in the same unit cell. To create the core holes, PAW potentials with one removed 3d (1s) electron were used for the In 3d (O 1s/C 1s) shifts. The charge neutrality of the computational cell was maintained by employing a jellium background.^36,37^ Bader charges were calculated using the code developed by the Henkelman group.^38−41^ Differential adsorption energies, Δ*E*_diff_, of molecules on the surface were calculated as
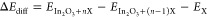
1where *E*_In_2_O_3_+*n*X_ and *E*_In_2_O_3_+(*n*–1)X_ are the total energies of the In_2_O_3_(111) surface slab with *n* adsorbed molecules and a slab with *n* – 1 adsorbed molecules, respectively. *E*_X_ is the energy of the molecule in the gas phase, which was computed at the Γ point in a simulation box of 15 Å side length.

## Results

In the following sections, we present (i) the characterization of the different surface terminations of In_2_O_3_(111) that will be used later to investigate CO_2_ adsorption, (ii) the adsorption of possible CO_2_ reduction reaction intermediates (formic acid and methanol) on stoichiometric In_2_O_3_(111), which we also use as benchmarks for the C 1s CLS, and (iii) the results for CO_2_ adsorption on the stoichiometric, reduced, and hydroxylated surface terminations of In_2_O_3_(111), respectively. In all sections, data from both the experimental XPS and computational CLS are used to explore the structure and behavior of the In_2_O_3_(111) surfaces.

### XPS Fingerprints of the Surface Terminations of In_2_O_3_(111)

**Figure 1:**
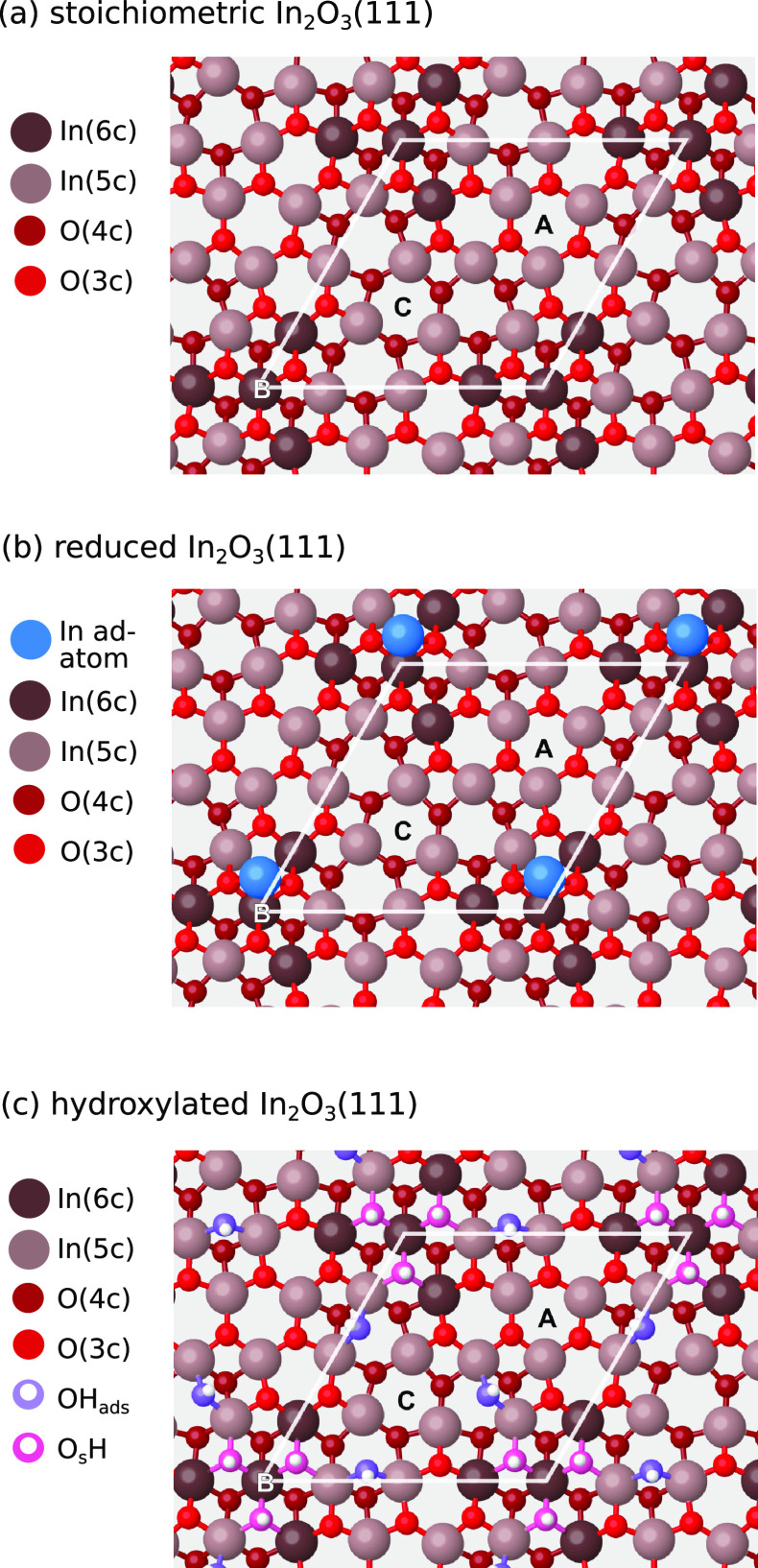
Top view of the first O–In–O trilayer of the (a) stoichiometric, (b) reduced, and (c) hydroxylated In_2_O_3_(111) surface termination. The 6- and 5-fold-coordinated In atoms are shown in dark brown and beige, respectively, whereas the O atoms occupying sites above and below the indium layer are shown in bright and dark red, respectively. On the hydroxylated surface, the O atoms of the OH groups are pink for O atoms belonging to the oxide lattice (O_s_H) and purple for the O atom originating from the dissociated water molecule (OH_ads_). In adatoms are blue. Note that the adatom configuration depicted here is the 2-fold-coordinated, which is isoenergetic with the structure depicted in ref (13). The surface cell is indicated with white lines.

**Figure 2:**
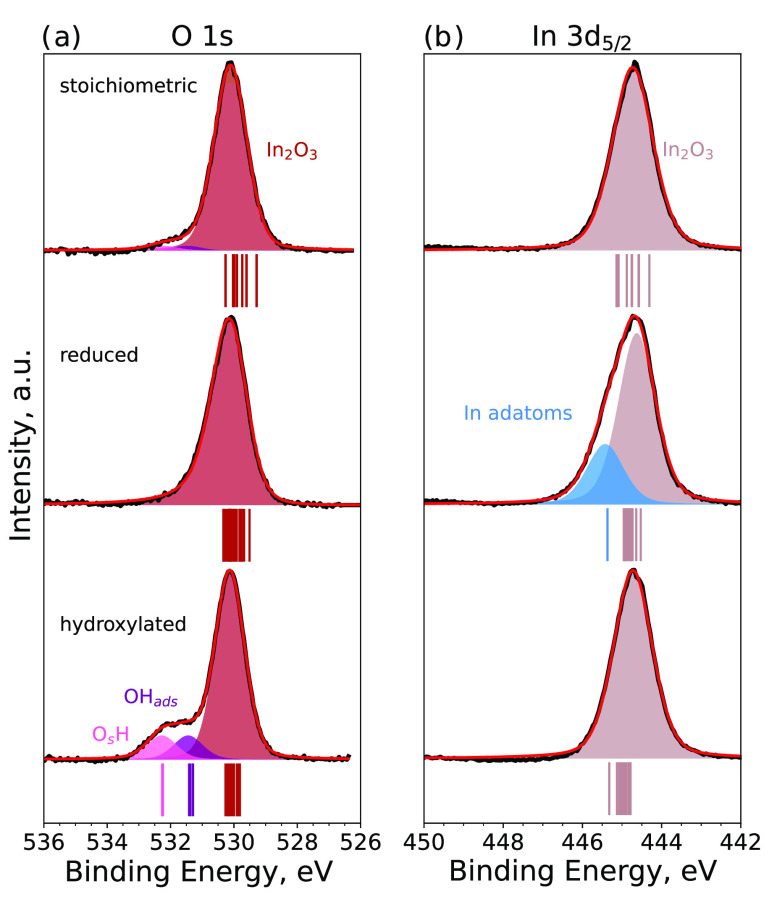
Experimental O 1s and In 3d_5/2_ core-level spectra of the three different In_2_O_3_ surface terminations. The spectra were background-subtracted and normalized to the intensity of the main peak. Calculated CLSs are reported as vertical lines below the experimental spectra, and the color coding is the same as that in the corresponding structures (Figure 1).

Figures 1a–c show the atomic structures of the stoichiometric, reduced, and hydroxylated In_2_O_3_(111) surface terminations, respectively, as determined in our DFT calculations. The structures agree with previous studies of these surface terminations.^12,18,19^ The experimental preparation of the surface terminations is described in the Experimental and Computational Methods section. Figure 2 shows the XPS spectra of O 1s and In 3d_5/2_ for different surface terminations. The spectra were background-subtracted and normalized to the intensity of the main peak. Details of the line shapes and background functions are listed in Table S1.

The spectra of stoichiometric In_2_O_3_(111) show a main peak at 530.1 eV in the O 1s core level, which corresponds to In_2_O_3_(111), and a small contribution of residual hydroxyl groups at higher binding energies. The In 3d_5/2_ core level shows a single symmetric peak at 444.7 eV. Contributions from differently coordinated atoms, or a surface CLS, could not be resolved experimentally by varying the photon energy and the electron emission angle. The CLSs obtained from DFT calculations are indicated below each XPS spectrum; the different lines show the results for the individual In and O atoms on the surface. The difference in atomic coordination results only in small shifts of the binding energies.

Reducing In_2_O_3_(111) can, in principle, lead to the formation of either oxygen vacancies or In adatoms. Previous scanning tunneling microscopy (STM) studies on In_2_O_3_(111) have reported that thermal reduction of the In_2_O_3_(111) surface results in the formation of an ordered array of In adatoms with one adatom per unit cell.^12^

The experimental O 1s core-level spectrum of thermally reduced In_2_O_3_(111) in Figure 2 shows a slight asymmetry toward higher binding energies. This observed asymmetry in O 1s could result either from changes in the electronic structure of the In_2_O_3_ surface to a more metallic nature or from the adsorption of a small number of oxygen-containing molecules from the background gas, such as small amounts of water. The In 3d_5/2_ shows a strong asymmetry toward higher binding energies, which originates from the formation of a new component in the XPS spectrum. The new component has a binding energy of 445.4 eV, and the bulk In_2_O_3_ shifts by 0.1 to 444.6 eV due to band-bending effects (see below). It is tempting to assign the component at higher binding energy to the In adatoms previously observed for the reduced surface.^12^ To validate this assignment, we calculated the relative binding-energy shift of In adatoms on the surface. We considered In adatoms placed at three different 3-fold symmetric sites on In_2_O_3_(111), labeled as A, B and C in Figure 1. The relative stabilities, Bader charges, and all In 3d CLSs of the In adatoms on these sites calculated with the PBE exchange-correlation functional are reported in Table S8. Our calculated adatom stabilities are in complete agreement with previous DFT calculations performed with another implementation of the DFT equations.^12^

The most stable site for In adatoms is the B site, where the adatom can coordinate to three or two O atoms. The structures are found to be nearly energetically degenerate (3 meV difference), suggesting that the adatom can move between the sites, even at low temperatures. The In 3d CLSs were calculated for all In atoms in the first O–In–O trilayer for stoichiometric and reduced In_2_O_3_(111). The In 3d shifts of the pristine surface cover a range of approximately 1 eV, with 6-fold (5-fold)-coordinated In cations having negative (positive) shifts with respect to the bulk. The In adatoms on the reduced surface show a positive shift of 0.8 eV compared with the other surface In atoms. This experimentally observed shift is very close to the calculated 0.7 eV shift for the adatom at the 2-fold-coordinated sites. This confirms that the In adatoms occupy the B site when In_2_O_3_(111) is reduced, which is in agreement with the previous STM and DFT studies.^12^

We observed that the reduction of the surface causes band bending at the surface, which results in binding-energy shifts for all core levels. The effect of the band bending can be quantified from the position of the VBM by calibrating the spectra to the Fermi level of a gold foil mounted next to the sample at room temperature. The VBM is at 3.0 eV for the stoichiometric and hydroxylated surfaces and at 3.2 eV for the reduced surface with In adatoms. A downward band bending of 0.5 eV has previously been reported for In_2_O_3_(001)^42^ between the stoichiometric and reduced surface termination. The obtained band gap for In_2_O_3_(111) is close to the band gap of single-crystalline In_2_O_3_, which has been reported to be at 2.93 ± 0.15 eV and 3.02 ± 0.15 eV for the cubic bixbyite and rhombohedral polymorphs, respectively.^43^

In the hydroxylation experiment with H_2_O shown in Figure 2, a new component appears in the O 1s spectrum at higher binding energies relative to the lattice oxygen. The shoulder can be deconvoluted into two features with binding energies of 531.5 and 532.3 eV, which correspond to binding energy shifts of +1.3 and +2.1 eV, respectively.

The DFT calculations show that hydroxylation of In_2_O_3_(111) by water is energetically preferred. The adsorption energy of a single water molecule is −0.74 eV. Upon adsorption, the water molecule can easily dissociate at the B site with a low barrier of 0.05 eV,^13^ and an exothermic reaction energy of −0.57 eV. Upon dissociation, two hydroxyl (OH) groups are formed on the surface, one is the OH fragment from water, OH_ad_, which binds to the In cations on the surface OH_ads_, and the other is formed as the proton from water binds to an O atom on the In_2_O_3_(111) surface, O_s_H. The dissociated water molecule adsorbs with adsorption energy of −1.31 eV. There are three equivalent sites close to the B site where water can adsorb dissociatively, and the effect of coverage on the adsorption energy is modest. Further adsorption of water takes place nondissociatively at the C site with lower binding energies compared to the dissociative adsorption to the B site. The O 1s and In 3d CLSs of the two OH groups were calculated for the structure containing three dissociated water molecules (Figure 1c). The CLSs of the O 1s atom (Figure 2) show that the OH groups give rise to characteristic peaks at higher binding energies with respect to the other surface O atoms. The average computed O 1s CLS is 2.2 eV for the three O_s_H groups and 1.3 eV for the three OH_ads_ groups. The computed CLSs for the two types of OH groups are in excellent agreement with the experimental XPS data (1.3 and 2.1 eV, respectively). The calculated O 1s CLS for molecularly adsorbed H_2_O on the In_2_O_3_(111) surface is over 3 eV with respect to the bulk.^14^ The absence of a strongly shifted peak in the O 1s XPS spectra supports the assessment that only dissociated water is present on the surface and is in agreement with the previous STM study of water on In_2_O_3_(111), which showed that it is possible to achieve a coverage of three water molecules per In_2_O_3_(111) unit cell at room temperature.^19,44^

### Methanol and Formic Acid on Stoichiometric In_2_O_3_(111)

**Figure 3:**
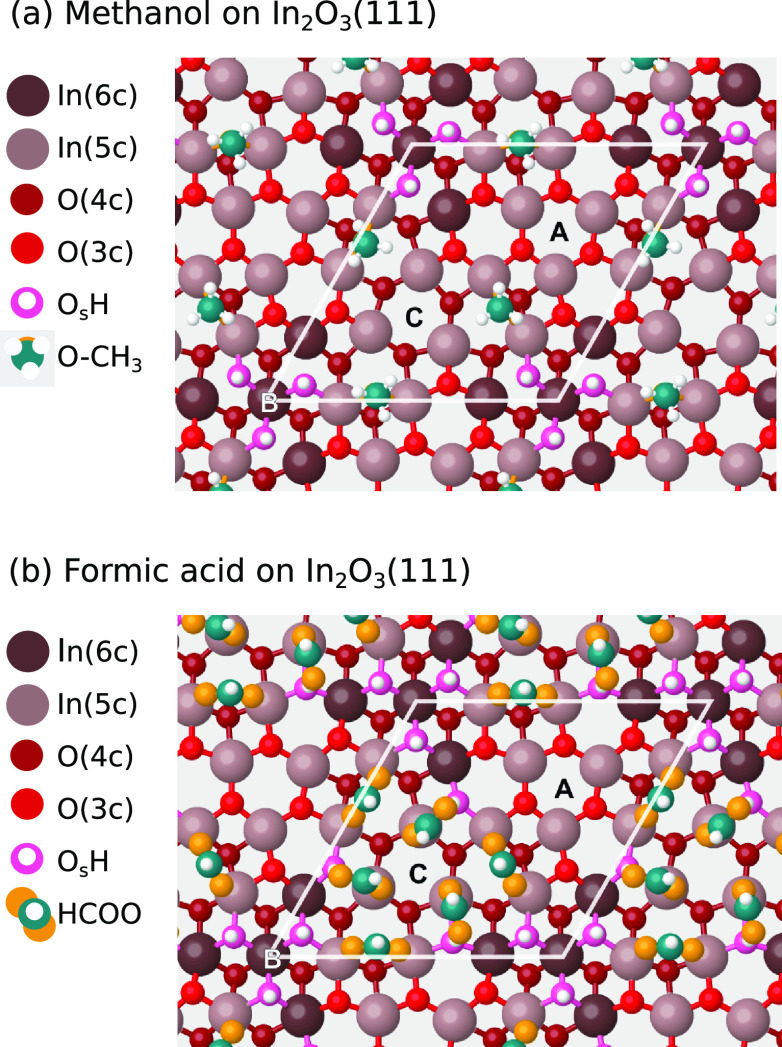
DFT-relaxed structures of (a) methanol and (b) formic acid on In_2_O_3_(111). C atoms of the HCOOH and methanol molecules are teal, whereas their O atoms are orange. In (a), the O atom of the O-CH_3_ is hidden by the C atom above it.

To study how the methanol product and possible reaction intermediate formic acid bind to the surface, their adsorption on stoichiometric In_2_O_3_(111) was studied experimentally and computationally. Figure 3a shows the atomic configuration of methanol on stoichiometric In_2_O_3_(111). Methanol was determined to preferably adsorb dissociatively on In_2_O_3_(111), forming H and O–CH_3_ (with Bader charges of +0.63 e and −0.70 e, respectively) pairs around the B site, preferring the same adsorption sites as those of dissociated water. The B site can accommodate three such pairs, which have very strong adsorption energies in the range of −1.2 to −1.0 eV. Achieving higher coverages requires the methanol to adsorb nondissociatively around the C site, binding to an In cation through its O atom. These methanol molecules have adsorption energies of only −0.5 to −0.4 eV, which suggests that only coverages of up to three methanol molecules per unit cell are achieved at room temperature.

**Figure 4:**
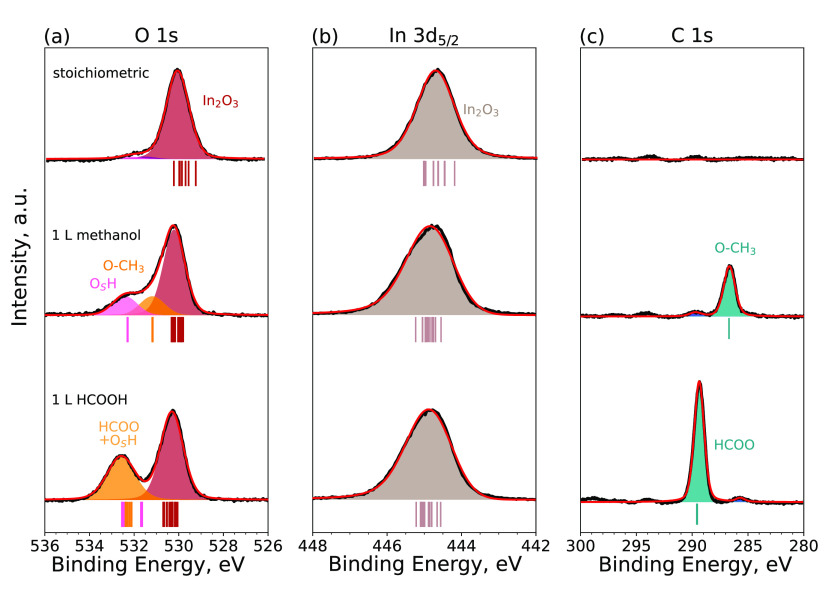
Experimental O 1s, In 3d_5/2_, and C 1s core-level spectra for methanol and formic acid adsorbed on In_2_O_3_(111). Reference spectra of the bare stoichiometric surface are provided. The calculated CLSs are indicated with vertical lines below the experimental spectra. The color coding of the lines is the same as the coloring of the atoms in the structural models (Figure 3b).

The O 1s, In 3d_5/2_, and C 1s spectra of the methanol-covered surfaces are shown in Figure 4a–c, respectively, along with the pristine In_2_O_3_(111) for comparison. After methanol adsorption, the XPS spectra show two new peaks in the O 1s core level at the respective binding energies of 531.2 and 532.4 eV. These peaks can be assigned to the O–CH_3_ fragment and the protonated O atom at the B site, O_s_H. The shift between the peaks is calculated as 1.12 eV, which is in nice agreement with the experimental value of 1.2 eV. In the C 1s spectrum, the O–CH_3_ groups result in a peak at 286.7 eV. The experimental In 3d_5/2_ spectrum shows an asymmetry to higher binding energy after the adsorption of methanol. This is qualitatively consistent with the calculated In 3d CLS: contributions from surface In atoms are found at slightly higher binding energies than those of the pristine surface. The asymmetry originates from the superposition of the bulk signal (at lower binding energies) and the signal of the surface In atoms. Interestingly, the highly coordinated In atoms of site B give the most positive CLS on the methanol-covered surfaces, whereas on the pristine surface, they exhibit a mildly negative CLS with respect to the bulk In atoms in the middle of the slab.

Additionally, we studied the adsorption of formic acid on the stoichiometric In_2_O_3_(111). The DFT calculations show that three formic acid molecules can adsorb dissociatively as a HCOO and H (with Bader charges of −0.75 e and +0.64 e, respectively) pairs around the B site, with the HCOO fragment in a bridging configuration between two In cations similar to the methoxy groups. The next three HCOOH molecules adsorb dissociatively around the C site: one O atom of the HCOO fragment binds to an In cation, and the other O atom coordinates to the nearby-adsorbed H atom (Figure 3). Similarly to the case of methanol and water, adsorption around the C site is less favorable than around the B site. However, the adsorption of HCOOH at site C is exothermic relative to the gas phase. This indicates that In_2_O_3_(111) can accommodate six formic acid molecules per unit cell at room temperature. The differential adsorption energies of these molecules are displayed in Figure S4 in the SI.

XPS spectra of formic acid on In_2_O_3_(111) are displayed in Figure 4. The O 1s spectrum shows an additional peak at 532.6 eV with a large full width at half maximum (fwhm) of 1.5 eV, indicating that multiple components contribute to this peak. After the adsorption of formic acid, In 3d_5/2_ core level shows increased asymmetry, similar to the methanol case. The C 1s spectrum shows a peak at 289.3 eV.

The computed O 1s CLS for HCOOH on the surface shows three groups of peaks. The surface O atoms that do not take part in the HCOOH adsorption show the lowest relative shifts. The O_s_H groups around site C have an average shift of 1.4 eV, whereas the HCOO and O_s_H groups around site B have shifts ranging from 1.8 to 2.2 eV. The computed O 1s shifts are in fair agreement with the O 1s XPS spectrum. Similar to methanol adsorption, the In 3d CLS shows that the surface In cations are shifted to slightly higher binding energies than those on the pristine surface.

The C 1s CLS of HCOOH was computed relative to the C 1s CLS of the reference O–CH_3_ group (see the SI for a detailed discussion). The calculations were performed by placing a dissociated methanol molecule in the same unit cell with formic acid and calculating the total energy with a core hole on each C atom. The CLSs were calculated for two separate cases, where the HCOO and H pair is bound to either the B site or the C site. The HCOO fragment at the B site has a shift of 2.78 eV, while HCOO bound to the C site has a shift of 2.82 eV, relative to O–CH_3_. The average relative CLS of the two HCOO groups and the O–CH_3_ group is close (2.8 eV) to the experimentally observed difference in the binding energy (2.6 eV) of the HCOOH and the O–CH_3_ C 1s peaks. Further analysis on the effect of the binding configuration, exchange-correlation functional, initial and final state effects, and surface coverage on calculated C 1s CLSs is presented in the SI.

The number of formic acid molecules per unit cell can be estimated from the area of the C 1s peak using the peak intensity of the O–CH_3_ peak as a reference, under the assumption that this saturated surface is covered by three dissociated methoxy molecules. Based on this assumption and the support from the DFT-calculated adsorption energy trends (Figure S4), we can conclude that six formic acid molecules can adsorb per unit cell, as illustrated in Figure 3b.

### CO_2_ Adsorption on Different Surface Terminations of In_2_O_3_(111)

**Figure 5:**
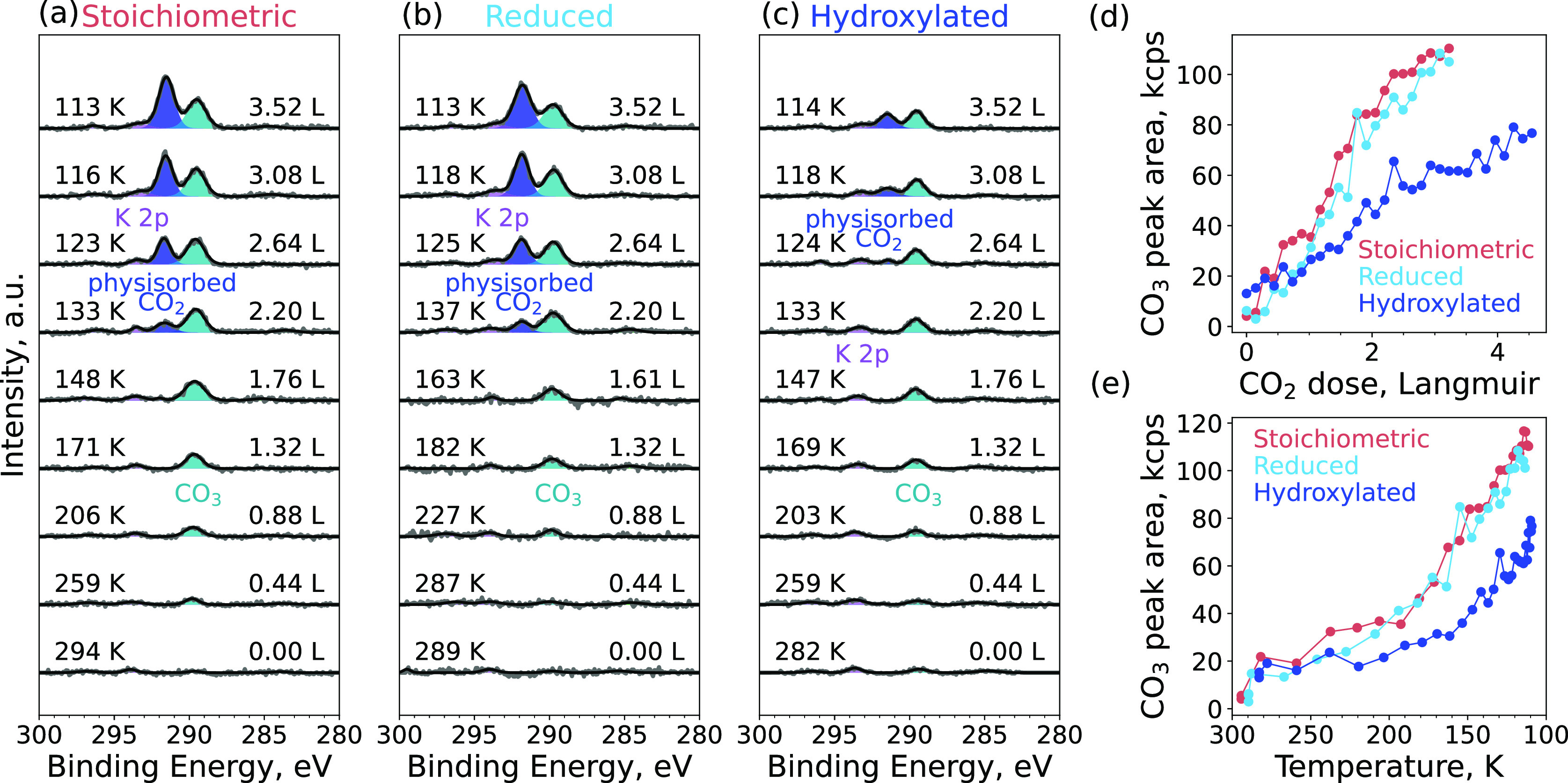
Measurements of the CO_2_ adsorption during sample cooling. C 1s core level of the (a) stoichiometric, (b) reduced, and (c) hydroxylated surfaces during CO_2_ adsorption at 5 × 10^–9^ mbar of CO_2_, while the samples are cooled from room temperature to 100 K. The numbers on the left-hand side of every panel give the temperature in Kelvin as the samples are cooled, and the numbers on the right-hand side of the panel give the CO_2_ dose in langmuir. (d) Area of the CO_3_ peak as a function of the CO_2_ dose in langmuir. (e) CO_3_ peak area as a function of the sample temperature.

The adsorption of CO_2_ was monitored experimentally on the three different terminations of the In_2_O_3_(111) surface discussed above. The surfaces were exposed to 5 × 10^–9^ mbar of CO_2_ while being cooled from room temperature to 100 K. We chose this experimental route to minimize the adsorption of H_2_O from the background that would otherwise occur when first cooling the sample and later dosing CO_2_. Figure 5a–c show the C 1s spectra of the stoichiometric, reduced, and hydroxylated In_2_O_3_(111) during CO_2_ adsorption, respectively. On all three surfaces, CO_2_ adsorption results in the development of an XPS feature at 289.7 ± 0.1 eV. Its position is consistent with the formation of carbonate (CO_3_).^45^ The peak becomes visible on all three surfaces at temperatures of around 200 K and grows as the surfaces are cooled. In comparison, the peak growth is slower on the hydroxylated surface than on the other two surface terminations, indicating that the hydroxyl groups on this surface hinder the adsorption of CO_2_. Conversely, adsorption on the reduced surface proceeds in a manner similar to that on the stochiometric surface, showing that In adatoms do not affect the CO_2_ adsorption. Figure 5d,e show the peak area of the carbonate peak as a function of the amount of dosed CO_2_ and temperature, respectively. A new peak appears as the surfaces are cooled to 140 K. This peak has a binding energy of 291.7 eV, which originates from physisorbed CO_2_.^45^ Again, the growth of this peak is considerably slower on the hydroxylated surface.

**Figure 6:**
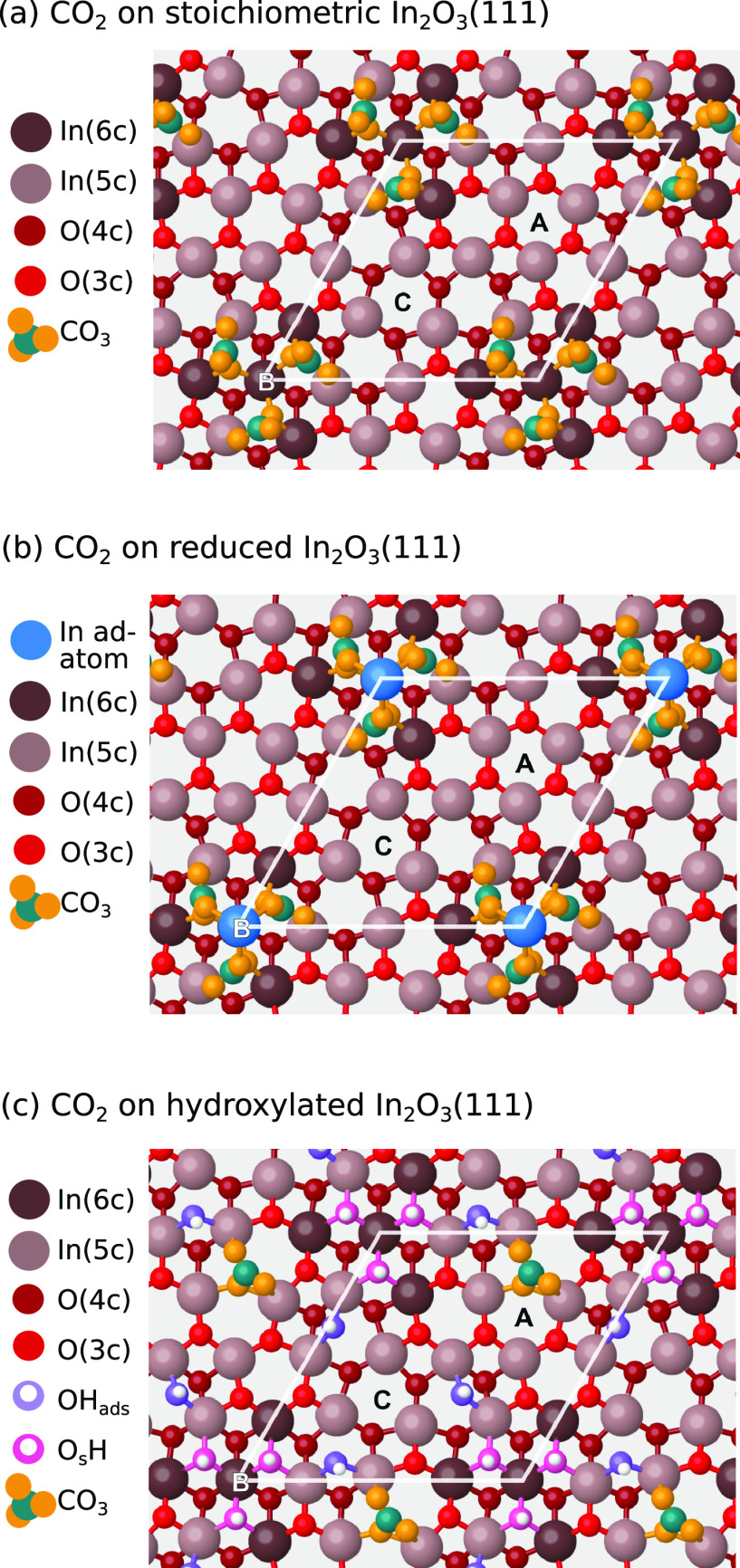
DFT-relaxed structures of CO_2_ adsorption on (a) stoichiometric, (b) reduced, and (c) hydroxylated In_2_O_3_(111). C atoms are teal, while the O atoms forming the CO_3_ carbonate species are orange.

We performed DFT calculations to identify the adsorption configuration of CO_2_ on the differently terminated In_2_O_3_(111) surfaces. The resulting structures are illustrated in Figure 6. On the pristine surface, the CO_2_ molecule can adsorb only by binding to a surface oxygen as a bent carbonate (CO_3_) species with a Bader charge of −1.46 e. The adsorption is preferred on the undercoordinated O atoms around the B site (Figure 6a). Here, the adsorption energy is −0.9 eV. The adsorption energy is lowered when more than one CO_2_ molecule is adsorbed at the B site, and once all three O atoms are occupied, additional CO_2_ adsorbs close to site A instead. The adsorption energy of additional CO_2_ at site A is weak (−0.3 to −0.1 eV), which indicates that, at most, three CO_2_ molecules can adsorb as carbonate on the pristine In_2_O_3_(111) surface. The calculated C 1s CLS of the carbonate is 3.0 eV with respect to the methoxy group. The relative shift is in good agreement with the experimentally observed binding energy difference of 3.0 eV between the methoxy and carbonate C 1s XPS peaks.

On the hydroxylated surface, the O atoms close to site B have all been converted to hydroxyl groups. The OH_ads_ and O_s_H pair bind stronger to the site than CO_2_ and preferably occupy adjacent sites.^19^ It is energetically unfavorable to displace a hydroxyl group and form a carbonate; thus, CO_2_ adsorption is blocked by water at the B site. The only sites left available for CO_2_ adsorption are the O atoms around site A, where the adsorption energy of a single CO_2_ is −0.7 eV. The adsorption of additional CO_2_ is less favorable with −0.5 and –0.4 eV for the second and third molecules, respectively. Interestingly, the adsorption of carbonate at site A is more favorable when site B hosts water than when it hosts other carbonate species. Unlike dissociated water, the formation of a carbonate requires a charge transfer from the surface to the adsorbate; thus, hydroxylated In_2_O_3_(111) can only accommodate one carbonate atoper unit cell.

As an alternative to adsorbing as a carbonate, CO_2_ could, in principle, react with one of the O_s_H or OH_ads_ groups on the hydroxylated surface to form formate (HCOO^–^) or bicarbonate (HCO_3_^–^). The C atom of the formate species has a calculated C 1s CLS of 3.0 eV with respect to the methoxy group peak (similar to the value of 2.9 eV of C 1s of carbonate species; see above), which would also be in very good agreement with the XPS data. However, adsorption is highly endothermic (approximately 3 eV) and thus unfavorable. Bicabonate species are more stable than formate; however, the calculated C 1s CLSs of all considered HCO_3_^–^ configurations are strongly shifted by approximately 4 eV to higher binding energy with respect to the methoxy peak. Therefore, we propose that CO_2_ adsorbs as a carbonate on the hydroxylated surface.

On reduced In_2_O_3_(111), we find that CO_2_ can adsorb as a carbonate in essentially the same geometry as on the pristine surface, but additionally coordinating to the In adatom. A single carbonate pushes the adatom off-center of the B site so that it preferably occupies the 2-fold coordinate site; however, the addition of more CO_2_ pushes the adatom back to the central position. The carbonate adsorption energy is more exothermic than that on the pristine surface, and it is possible to populate all three B-site O atoms simultaneously.

It is possible to estimate the number of molecules per unit cell by comparing the peak area of adsorbed CO_3_ in the experimental C 1s spectrum to the peak area of adsorbed methanol. With a methanol coverage of three molecules per unit cell, the CO_2_ coverage on the stoichiometric and reduced surfaces corresponds to approximately 1.7 molecules per unit cell and to one molecule per unit cell on the hydroxylated surface. While the experimentally observed coverage on the hydroxylated surface is in agreement with the DFT calculations, the experimental coverage on the stoichiometric and reduced surfaces of 1.7 molecules per unit cell is lower than the three molecules per unit cell predicted by DFT calculations. We speculate that the discrepancy is due to the adsorption of water from residual gas in the vacuum chamber as the samples are cooled. The adsorbed water molecules effectively block adsorption sites for CO_2_ as they do on the hydroxylated surface and thus lower the CO_2_ coverage observed in the experiments.

**Figure 7:**
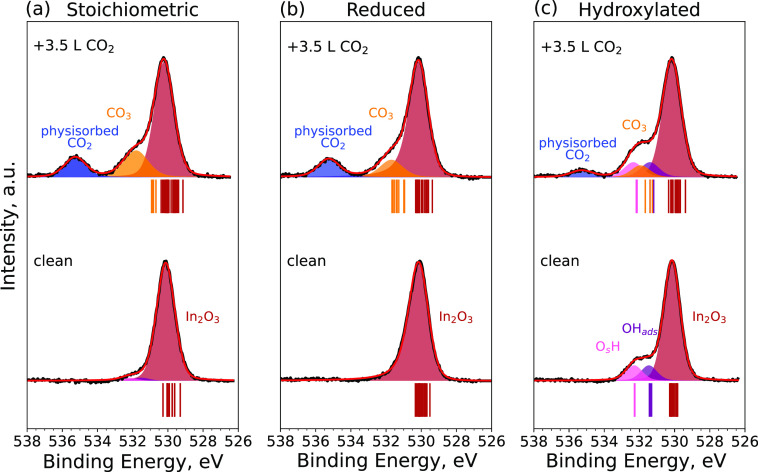
O 1s core-level spectra of the (a) stoichiometric, (b) reduced, and (c) hydroxylated In_2_O_3_(111) surfaces at room temperature prior to the adsorption of CO_2_ (bottom) and after the adsorption of 3.5 langmuir of CO_2_ at 5 × 10^–9^ mbar (top) at a final temperature of approximately 100 K. The corresponding calculated CLSs are indicated with vertical lines under each spectrum, and their colors match the coloring of the atoms in the atomic structure figures.

**Figure 8:**
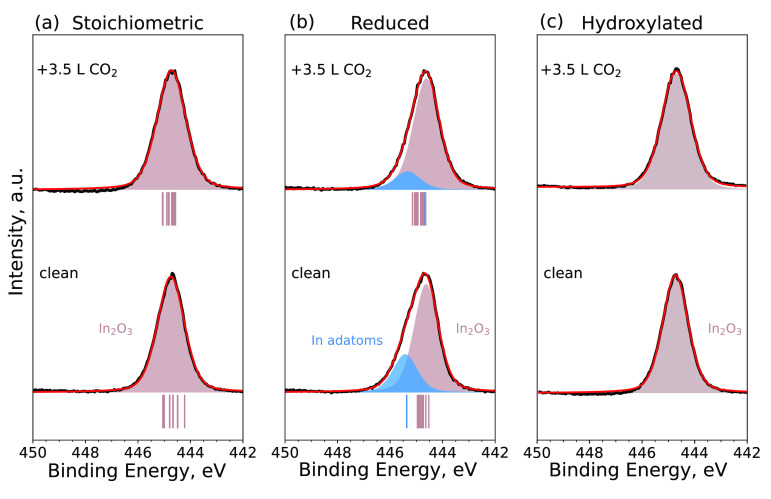
In 3d_5/2_ core-level spectra of the (a) stoichiometric, (b) reduced, and (c) hydroxylated In_2_O_3_(111) surfaces at room temperature prior to the adsorption of CO_2_ (bottom) and after the adsorption of 3.5 langmuir of CO_2_ at 5 × 10^–9^ mbar (top) at a final temperature of approximately 100 K. The corresponding calculated CLSs are indicated with vertical lines under each spectrum.

Figures 7 and 8 show the experimental XPS spectra of the O 1s and In 3d_5/2_ core levels of the three surface terminations before and after CO_2_ adsorption, respectively. To support the experimental data, we also performed DFT calculations for CO_2_ adsorbed in a carbonate configuration on three In_2_O_3_(111) surface terminations. The CLSs in Figures 7 and 8 were calculated at a coverage of three carbonates per unit cell for the pristine and reduced surfaces and one carbonate per unit cell for the hydroxylated surface. For the stoichiometric In_2_O_3_(111), a minor broadening of the In_2_O_3_ peak in the O 1s and In 3d core levels is observed after CO_2_ adsorption. The adsorption of CO_2_ appears to diminish the peak from the In adatoms by 55% in the In 3d core level, whereas no change is observed in the O 1s spectrum. For the hydroxylated surface, the O 1s and In 3d peaks broaden after CO_2_ adsorption. The DFT calculations show that the O atoms of the carbonate (CO_3_) are positively shifted with respect to a bulk O on the pristine surface; however, the shifts are not as clearly distinguishable from the main surface O peak as determined experimentally. On the reduced surface, the O 1s CLSs of the CO_3_ species and the surface O atoms are more separated and agree well with the experimental spectrum. Finally, on the hydroxylated surface, the CO_3_ CLSs are partially overlapping with the OH_ads_ peak; however, the computed CLSs are still in good qualitative agreement with the observed XPS spectra. Calculated In 3d CLSs show very little change upon CO_2_ adsorption for the pristine and hydroxylated surfaces. On the reduced surface, CO_2_ shifts the peak of the In adatoms toward the bulk In_2_O_3_, agreeing very well with the experimentally observed suppression of the adatom peak upon CO_2_ adsorption.

## Discussion

We have presented the XPS spectra of stoichiometric, reduced, and hydroxylated In_2_O_3_(111), as well as changes upon the adsorption of methanol and formic acid on stoichiometric In_2_O_3_(111). The experimentally observed XPS spectra are overall in good agreement with the presented DFT CLS calculations. Thus, the experimental spectra and calculated CLSs are important references for experiments performed at elevated pressures and temperatures.

Additionally, we studied the adsorption of CO_2_ on different surface terminations of In_2_O_3_(111). The experiments showed that hydroxyl groups on In_2_O_3_(111) partially block the adsorption of CO_2_. Water has been reported to decrease the activity of CO_2_ hydrogenation for In_2_O_3_^11^ and other CO_2_ hydrogenation catalysts.^46^ Our previous DFT-based microkinetic models have also shown that OH can block surface sites of In_2_O_3_(110), which leads to a negative reaction order with respect to the partial pressure of water.^13^ The DFT calculations, presented in this article, indicate that water and CO_2_ preferably adsorb at the same surface site. The dissociative water adsorption to the site is stronger than the physisorption of CO_2_, and therefore the resulting hydroxyl groups can block CO_2_ from adsorbing as carbonate. Our computed C 1s CLS for carbonate closely matches the experimental shift relative to the methanol C 1s peak. The agreement between the experiments and computations was achieved by using the well-defined methanol and formic acid C 1s peaks as references. For the C 1s CLS, we find that including exact exchange by employing a hybrid functional is vital to achieving the correct relative shifts for C-containing adsorbates on In_2_O_3_(111).

The adsorption of CO_2_ on different catalysts for CO_2_ hydrogenation, such as CeO_2_, Rh, and CuZn, has been studied previously. CO_2_ has been reported to adsorb as a carbonate on CeO_2_ as well as on Zn deposited on Cu surfaces,^45,47^ while it was reported to dissociate on Rh^48^ and stepped Cu surfaces.^49^ The present measurements show that CO_2_ does not dissociate on In_2_O_3_(111) when adsorbed at a pressure of 5 × 10^–9^ mbar and temperatures below room temperature. We could not observe the formation of formate on the surface when CO_2_ was adsorbed on the hydroxylated surface. This suggests that higher pressures, temperatures, or additional gases are required to activate CO_2_ for the hydrogenation reaction. Our previous DFT studies suggest that the In_2_O_3_ surface is partially hydrogenated under typical reaction conditions,^14^ with kinetic studies on hydrogenated In_2_O_3_(110)^13^ supporting the notion that a hydrogenated surface forms the active site for the methanol synthesis from CO_2_. In contrast to the hydrogenated surface, hydroxylation by water does not result in a change in the oxidation state^14,50^ for the surface In atoms and does not facilitate the activation of CO_2_.

A detailed understanding of the adsorption of CO_2_ on In_2_O_3_ is an important step toward understanding the catalytic process of thermal CO_2_ hydrogenation over In_2_O_3_ catalysts on the atomic scale. In a previous study, the reaction mechanism of CO_2_ hydrogenation has been attributed to the interaction of reactants with oxygen vacancies.^17^ However, we have no evidence of the existence of these defects in the processes that we have studied so far.

**Figure 9:**
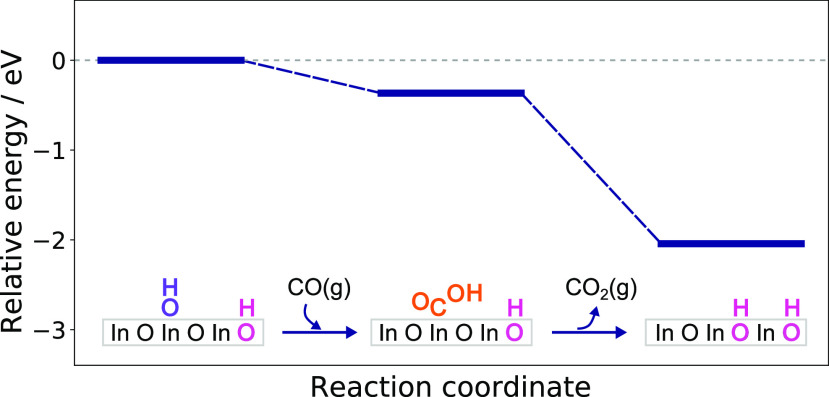
DFT-calculated energy profile for the WGS reaction between a surface O_ads_H group and a gas-phase CO.

The catalytic activity of In_2_O_3_ and CuZn increases when CO is added to the CO_2_ and H_2_ gas mixture.^11^ In this context, it is interesting to note that for CuZn catalysts it has been shown that the increased activity results from the removal of hydroxyl groups from the surface by CO via the WGS reaction.^51^ We speculate that a similar mechanism may occur for the In_2_O_3_ surfaces, explaining the promotional effect of adding CO to the CO_2_ and H_2_ gas feed. Without calculating barriers, we can evaluate the thermodynamic feasibility of the WGS reaction on In_2_O_3_(111) by considering the reaction between CO and a surface hydroxyl (Figure 9). It has been shown previously in the case of *m*-ZrO_2_(1̅11) that CO and OH cannot directly form formate in a single elementary step but instead react to form its structural isomer, carboxyl.^52^ Thus, we also consider the WGS reaction to proceed through the carboxyl group, which consequently dissociates into CO_2_ and a proton on the surface. Our thermodynamic analysis shows that the reaction is feasible on In_2_O_3_(111); therefore, it may be possible that CO can remove OH groups through the WGS reaction.

## Conclusions

We have studied different surface terminations of In_2_O_3_(111), stoichiometric, reduced (with In adatoms), and hydroxylated, and the adsorption of CO_2_ on these different surface terminations using XPS and DFT. Our results confirm the structure of the In adatom overlayer and the adsorption site of the hydroxyl groups reported in the literature. The experiments on CO_2_ adsorption showed that the In adatoms do not hinder the adsorption of CO_2_, while hydroxyl groups on the surface partially block the adsorption of CO_2_. The DFT calculations showed that CO_2_ does not dissociate and adsorbs as a carbonate on all of the studied surface terminations of In_2_O_3_(111).

## Supporting Information Available

Tables showing the line shapes, background shapes, and fwhm of the XPS spectra shown in the main text, comparison of stabilities, Bader charges, and In 3d CLSs of different adatom placements, comparison of stabilities and PBE/HSE06-calculated C 1s CLSs of different C-containing species on the hydroxylated In_2_O_3_(111) surface, plots of the adsorption energy as a function of the coverage for water, methanol, formic acid, and CO_2_ on the pristine In_2_O_3_(111) surface, and plot of the adsorption energies of CO_2_ on the pristine, reduced, and hydroxylated In_2_O_3_(111) surfaces as a function of CO_2_ (carbonate) coverage (PDF)
