# Supplementary material for: Effect of Different In2O3(111) Surface Terminations on CO2 Adsorption
Source: ACS Appl Mater Interfaces. 2023 Sep 13;15(38):45367–77. doi: 10.1021/acsami.3c07166 (PMC10540140; doi:10.1021/acsami.3c07166)
Supplement: Supplementary file 1 — am3c07166_si_001.pdf [file am3c07166_si_001.pdf]

Supplementary Information: Effect of Different $In_2O_3(111)$ Surface Terminations on CO_2 Adsorption

Sabrina M. Gericke,[*,†,@] Minttu M. Kauppinen,[*,‡,@] Margareta Wagner,[¶] Michele Riva,[¶] Giada Franceschi,[¶] Alvaro Posada-Borbón,[‡] Lisa Rämisch,[†] Sebastian Pfaff,[†] Erik Rheinfrank,[¶] Alexander M. Imre,[¶] Alexei B. Preobrajenski,[§] Stephan Appelfeller,[§] Sara Blomberg,[‖] Lindsay R. Merte,[⊥] Johan Zetterberg,[†] Ulrike Diebold,[¶] Henrik Grönbeck,[‡] and Edvin Lundgren[#]

†*Division of Combustion Physics, Lund University, 221 00 Lund, Sweden.*

‡*Department of Physics and Competence Centre for Catalysis, Chalmers University of Technology, 412 96 Göteborg, Sweden.*

¶*Institute of Applied Physics, TU Wien, 1040 Vienna, Austria.*

§*MAX IV Laboratory, Lund University, 221 00 Lund, Sweden.*

‖*Department of Chemical Engineering, Lund University, 221 00 Lund, Sweden.*

⊥*Department of Materials Science and Applied Mathematics, Malmö University, 205 06 Malmö, Sweden.*

#*Division of Synchrotron Radiation Research, Lund University, 221 00 Lund, Sweden.*

@*Contributed equally to this work*

E-mail: sabrina_maria.gericke@forbrf.lth.se; minttu.m.kauppinen@jyu.fi

XPS fitting parameters

The following section contains the fitting parameters used for the XPS spectra in the main text.

Table S1: Fitting parameters for the XPS spectra in Figure 2 of the main text.

Surface termination	Core level	Peak label	Binding energy (eV)	FWHM (eV)	Line shape	Background shape
hydroxylated	In $3d_{5/2}$	In_2O_3	444.7	1.2	SGL(30)	Shirley
	O 1s	In_2O_3	530.1	1.1	SGL(20)	Linear
	O 1s	OH_{ads}	531.4	1.1	SGL(20)	Linear
	O 1s	O_sH	532.3	1.1	SGL(20)	Linear
reduced	In $3d_{5/2}$	In_2O_3	444.6	1.1	SGL(30)	Shirley
	In $3d_{5/2}$	In adatoms	445.4	1.1	SGL(30)	Shirley
	O 1s	In_2O_3	530.2	1.2	LA(1,1.9,200)	Linear
stoichiometric	In $3d_{5/2}$	In_2O_3	444.7	1.2	SGL(30)	Shirley
	O 1s	In_2O_3	530.2	1.2	SGL(20)	Linear
	O 1s	OH_{ads}	531.4	1.1	SGL(20)	Linear
	O 1s	O_sH	532.3	1.1	SGL(20)	Linear

Table S2: Fitting parameters for the O 1s spectra in Figure 4(a) of the main text.

Surface termination	Core level	Peak label	Binding energy (eV)	FWHM (eV)	Line shape	Background shape
stoichiometric	O 1s	In_2O_3	530.2	1.2	SGL(20)	Linear
	O 1s	OH_{ads}	531.4	1.1	SGL(20)	Linear
	O 1s	O_sH	532.3	1.1	SGL(20)	Linear
methanol	O 1s	In_2O_3	530.2	1.1	SGL(20)	Linear
	O 1s	$O{-}CH_3$	531.2	1.3	SGL(20)	Linear
	O 1s	O_sH	532.3	1.3	SGL(20)	Linear
formic acid	O 1s	In_2O_3	530.2	1.1	SGL(20)	Linear
	O 1s	HCOO + O_sH	532.6	1.5	SGL(20)	Linear

Table S3: Fitting parameters for the In $3d_{5/2}$ spectra in Figure 4(b) of the main text.

Surface termination	Core level	Peak label	Binding energy (eV)	FWHM (eV)	Line shape	Background shape
stoichiometric	In $3d_{5/2}$	In_2O_3	444.7	1.2	SGL(30)	Shirley
methanol	In $3d_{5/2}$	In_2O_3	444.8	1.1	LA(1,10,450)	Shirley
formic acid	In $3d_{5/2}$	In_2O_3	444.7	1.2	LA(1,3,450)	Shirley

Table S4: Fitting parameters for the C 1s spectra in Figure 4(c) of the main text.

Surface termination	Core level	Peak label	Binding energy (eV)	FWHM (eV)	Line shape	Background shape
methanol	C 1s	O–CH_3	286.7	1.2	SGL(30)	Linear
formic acid	C 1s	HCOO	289.3	1.0	SGL(30)	Linear

Table S5: Fitting parameters for the C 1s spectra with high coverage in Figure 5(a), 5(b) and 5(c) of the main text.

Surface termination	Core level	Peak label	Binding energy (eV)	FWHM (eV)	Line shape	Background shape
stoichiometric	C 1s	physisorbed CO_2	291.7	1.2	SGL(30)	Linear
	C 1s	CO_3	289.7	1.5	SGL(30)	Linear
reduced	C 1s	physisorbed CO_2	291.7	1.2	SGL(30)	Linear
	C 1s	CO_3	289.6	1.5	SGL(30)	Linear
hydroxylated	C 1s	physisorbed CO_2	291.7	1.5	SGL(30)	Linear
	C 1s	CO_3	289.7	1.3	SGL(30)	Linear

Table S6: Fitting parameters for the O 1s spectra in Figure 7 of the main text for the spectra after CO_2 adsorption.

Surface termination	Core level	Peak label	Binding energy (eV)	FWHM (eV)	Line shape	Background shape
	O 1s	In_2O_3	530.2	1.3	SGL(20)	Linear
stoichiometric	O 1s	CO_3	531.8	1.1	SGL(20)	Linear
	O 1s	physisorbed CO_2	535.3	1.1	SGL(20)	Linear
	O 1s	In_2O_3	530.2	1.3	SGL(20)	Linear
reduced	O 1s	CO_3	531.7	1.6	SGL(20)	Linear
	O 1s	physisorbed CO_2	535.3	1.5	SGL(20)	Linear
	O 1s	In_2O_3	530.2	1.3	SGL(20)	Linear
	O 1s	OH_{ads}	531.4	1.3	SGL(20)	Linear
hydroxylated	O 1s	CO_3	531.9	1.3	SGL(20)	Linear
	O 1s	O_sH	532.4	1.3	SGL(20)	Linear
	O 1s	physisorbed CO_2	535.3	1.4	SGL(20)	Linear

Table S7: Fitting parameters for the XPS spectra in Figure 8 of the main text for the spectra after CO_2 adsorption.

Surface termination	Core level	Peak label	Binding energy (eV)	FWHM (eV)	Line shape	Background shape
hydroxylated	In $3d_{5/2}$	In_2O_3	444.7	1.3	LA(1.5,200)	Shirley
reduced	In $3d_{5/2}$	In_2O_3	444.6	1.1	SGL(30)	Shirley
	In $3d_{5/2}$	In adatoms	445.3	1.1	SGL(30)	Shirley
stoichiometric	In $3d_{5/2}$	In_2O_3	444.7	1.3	SGL(30)	Shirley

LEED images

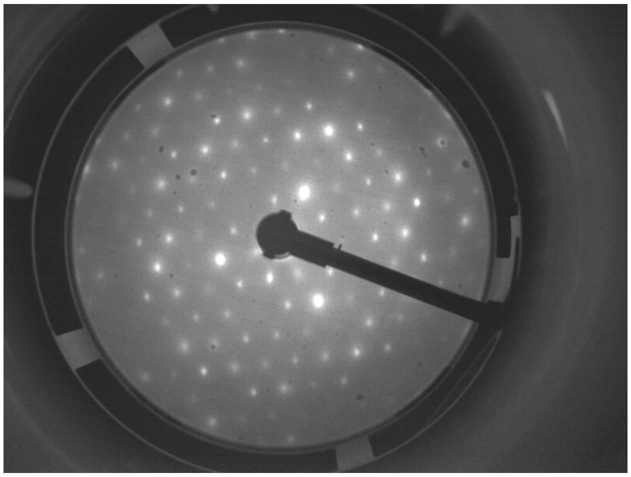

Figure S1: LEED image of the stoichiometric $In_2O_3(111)$ surface measured with 63 eV.

adatoms on reduced $In_2O_3(111)$

The relative stabilities and Bader charges of the In adatoms at different sites calculated at the PBE level of theory are given in Table S8. Geometries where the adatom binds to the A or C site are considerably higher in energy than at the B site.

The adatoms have In 3d core-level shifts (CLS) at higher binding energies with respect to the bulk indium atom (see Figure S3). Indeed, the adatoms should be shifted to higher binding energies compared to the bulk (and other low-coordinated surface indium atoms), due

Table S8: Stabilities and Bader charges of In adatoms on different $In_2O_3(111)$ high symmetry sites. The stabilities are calculated with respect to B_h, the high-symmetry 3-fold hollow site created by O atoms at the B-site. B_b is a bridge site between two of the O atoms. The Bader charges at the different sites are obtained as the difference between the number of Bader electrons and electrons on the neutral atom of the element.

site	relative stability /eV	Bader charge
A	+0.83	+0.75
B_b	+0.00	+0.75
B_h	0	+0.77
C	+1.38	+0.74

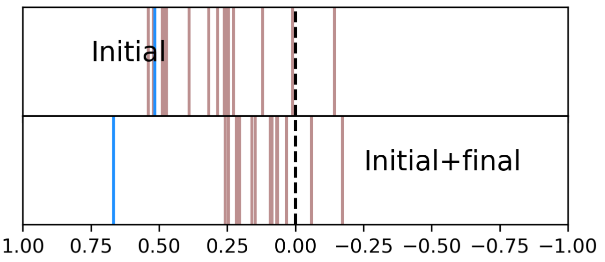

Figure S2: Surface In 3d core-level shifts for an adatom at B_b site of the $In_2O_3(111)$ surface calculated in the electrostatic initial state picture (top) and in the complete screening picture (bottom). The adatom CLS are shown in blue, while CLS from other surface In atoms are shown in brown. The dashed line shows the position of the bulk In.

to the adatoms experiencing lower Madelung potentials compared to the bulk.[S1,S2] However, final state effects arising from the electronic relaxation upon creating the core hole could still be important. To illustrate this, we obtained the In 3d CLS in the initial state picture by re-calculating the Kohn-Sham eigenvalues of the core states for the adatom bound at the B_b site. As can be seen in Fig. S2, the CLS for the adatom computed in the initial picture is not distinguishable from the other surface indium atoms. The inclusion of final state effects yields a larger positive shift for the adatom and less positive shifts for other surface indium atoms, allowing the shift from the adatom to be resolved.

The In 3d CLS of In atoms in the first O-In-O trilayer were calculated (including final state effects) for all adatom containing structures (Fig S3). All adatoms show a positive shift with respect to an In atom in the centre of the slab, shifted further than any other surface In atom.

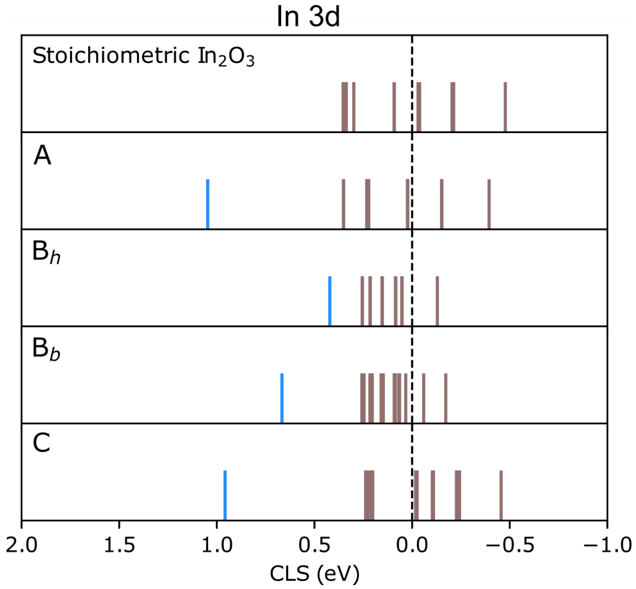

Figure S3: Surface In 3d CLS for stoichiometric and adatom containing $In_2O_3(111)$ surface. The adatom CLS are shown in blue, while CLS from other surface In atoms are shown in brown. The dashed line shows the position of the bulk In in each system.

Surface coverages of water, formic acid, and methanol

The effect of coverage on the adsorption energy of water, formic acid, and methanol were investigated on the stoichiometric $In_2O_3(111)$ in order to determine their saturation coverages. For each adsorbate, the most favourable adsorption sites were determined first for a single molecule in the unit cell. All three molecules prefer to adsorb at the B site dissociatively, formic acid having the strongest binding energy. The next best adsorption site for all molecules is around the C site, with water and methanol adsorbing molecularly to the site, while formic acid preferably donates a proton to a surface oxygen, binding in a tilted configuration. The structures with 1-3 molecules per unit cell were constructed by populating all three equivalent B sites one by one and relaxing the structures. Structures with 4-6 molecules per unit cell were constructed by taking the structure with all B sites occupied, adding molecules to site C one by one, and relaxing the obtained structures. The results

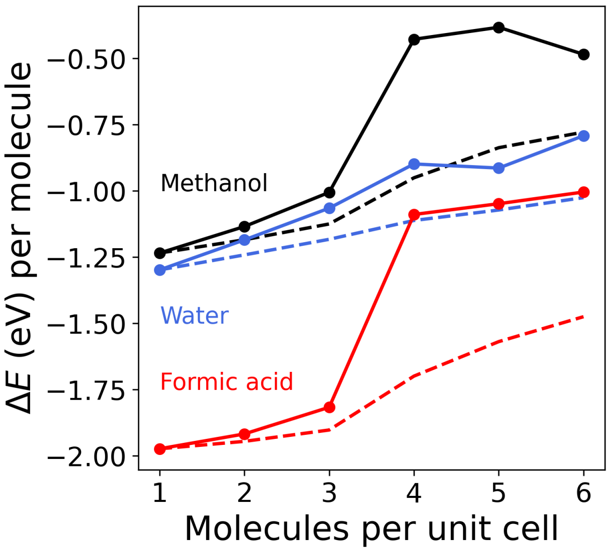

Figure S4: Differential (solid lines) and average (dashed lines) adsorption energies of water (blue), methanol (black), and formic acid (red) as a function of coverage.

(Fig. S4) show that the adsorption energy to the site B is only slightly lowered when more than one molecule is adsorbed around it. Differential adsorption energy to site C is much lower than site B, especially for formic acid and methanol, with increasing coverage having

only a small effect. Based on the adsorption energies, methanol and water should have a saturation coverage of 3 molecules per unit cell at room temperature. Formic acid adsorbs on the surface more strongly, and could achieve a saturation coverage of 6 molecules per unit cell.

Surface coverage of carbonate on stoichiometric, reduced, and hydroxylated $In_2O_3(111)$

Adsorption of CO_2 as a carbonate species was investigated on the stoichiometric, reduced, and hydroxylated terminations of $In_2O_3(111)$. On the stoichiometric surface, the CO_2 molecule binds to the oxygen atom around site B in a bent geometry. Incorporating three CO_2 molecules around the site is possible, with the differential adsorption energy decreasing upon addition of the second and third CO_2.

On the reduced surface, the CO_2 can adsorb at the B-site oxygen atoms, coordinating also to the adatom which is adsorbed at the center of the B site. The adsorption of up to three CO_2 molecules around site B on the reduced $In_2O_3(111)$ is more exothermic than on the stoichimetric $In_2O_3(111)$, indicating that the presence of the adatom does not block adsorption of CO_2 as carbonate.

Effect of exact exchange and OH coverage on O 1s binding energies

The O 1s CLS of hydroxyl groups formed on $In_2O_3(111)$ upon dissociative water adsorption were calculated at the PBE and HSE06 level of theory with one (low coverage) and three (saturation coverage) dissociated water in the unit cell (Fig. S6). At low coverage, the PBE/HSE06 calculated O 1s CLS with respect to a bulk oxygen are 1.23/1.26 and 1.95/1.99 eV for the OH_{ads} and O_sH groups, respectively. At the saturation coverage, the OH_{ads} and

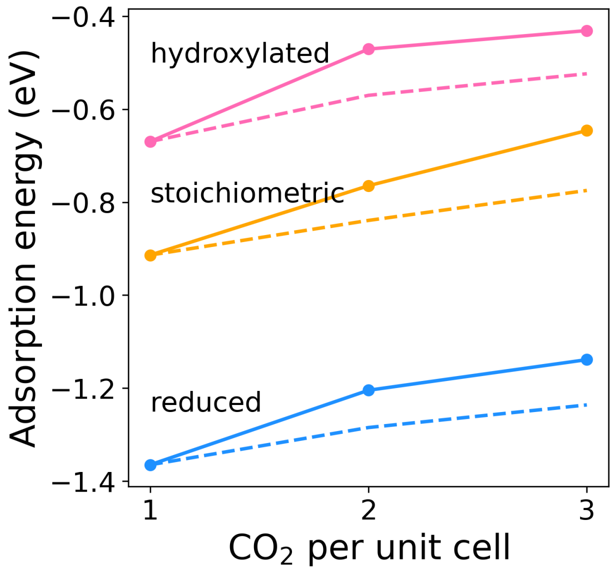

Figure S5: Differential (solid lines) and average (dashed lines) adsorption energies of carbonate as a function of coverage on stoichiometric (yellow), reduced (blue), and hydroxylated (pink) $In_2O_3(111)$.

O_sH groups give slightly different O 1s CLS, the average CLS computed using PBE/HSE06 being 1.28/1.40 and 2.17/2.30 eV, respectively. The PBE computed values at saturation coverage give the best agreement with experimental binding energy shifts (1.3 and 2.1 eV for OH_{ads} and O_sH, respectively). The PBE and HSE06 computed values at low coverage slightly underestimate the shifts with respect to experiments, especially for the O_sH species, while the HSE06 calculated values at saturation coverage slightly overestimate the shifts.

Carbon containing adsorbates on $In_2O_3(111)$

In order to support the peak assignment of the experimental XPS spectra of the CO_2 adsorption studies, the C 1s CLS of various possible adsorption structures (Fig. S7) have to be calculated. However, only relative CLS can be obtained within the applied methodology. Since there are no bulk carbon atoms in the stoichiometric In_2O_3 structure, there is no reference carbon atom in the system, unlike in the case of oxygen and indium. Therefore to provide a reference molecule on the surface that CLS of other adsorbates could be calculated

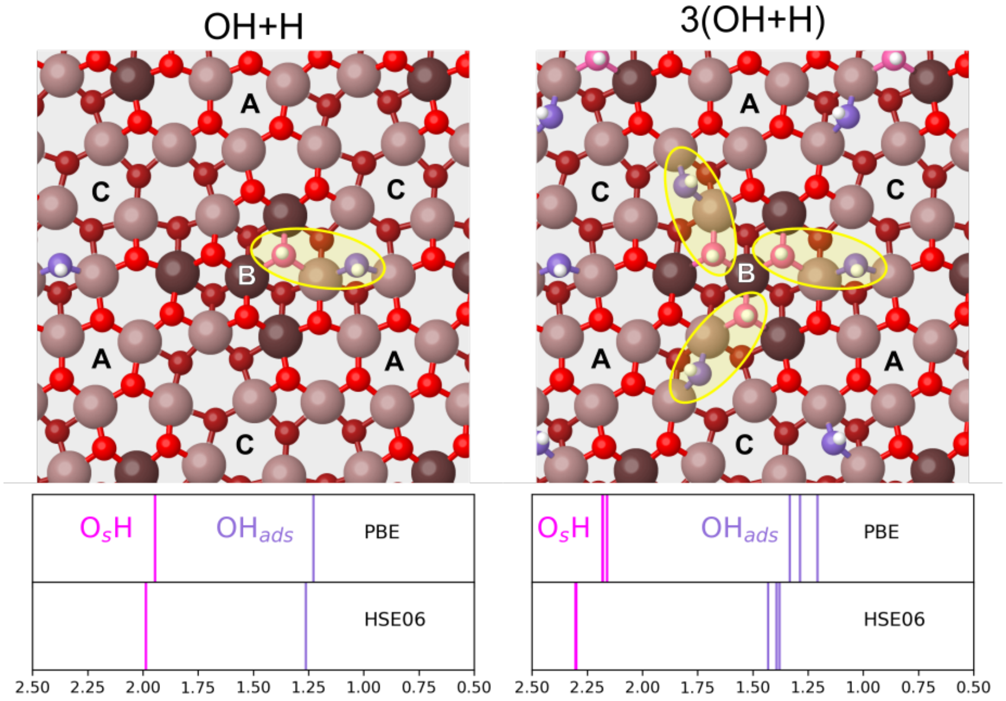

Figure S6: Optimised structures (top) and O 1s CLS (bottom) for hydroxyl group containing $In_2O_3(111)$ surfaces for two different coverages calculated with PBE and HSE06 functionals.

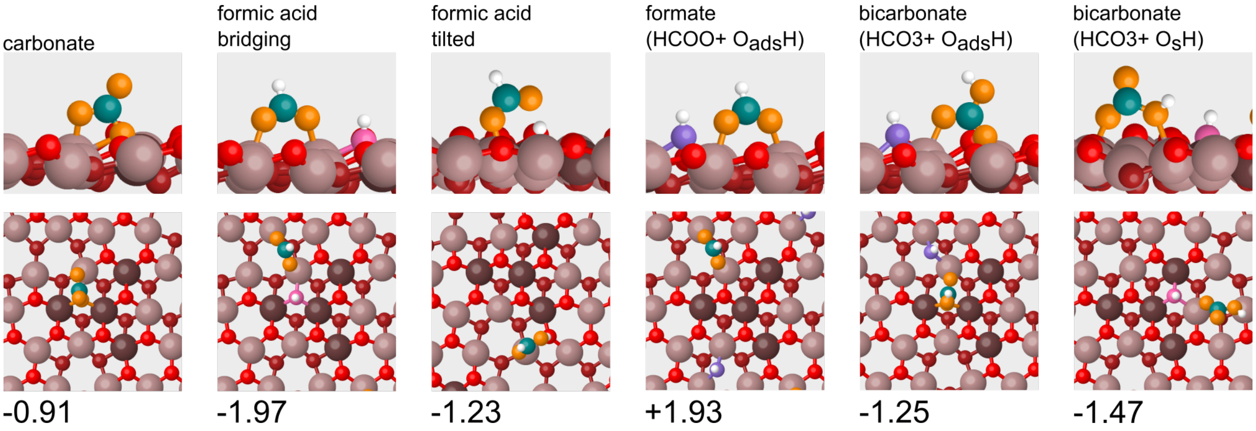

Figure S7: Optimised geometries and adsorption energies (in eV) of various carbon containing adsorbates on $In_2O_3(111)$. The adsorption energies are given with respect to the clean stoichiometric $In_2O_3(111)$ slab and CO_2 (carbonate), formic acid (formic acid), or CO_2 and H_2O (formate and bicarbonate) in the gas-phase.

with reference to, the adsorption of formic acid and methanol were studied experimentally on the stoichiometric $In_2O_3(111)$. Both molecules give clear peaks in the C 1s spectra, separated by 2.6 eV. To calculate the relative shift between formic acid and methanol, both molecules were placed on a stoichiometric $In_2O_3(111)$ slab in the same unit cell (Fig. S8). The relative C 1s core-level shift (ΔCLS), was then calculated with the gradient-corrected

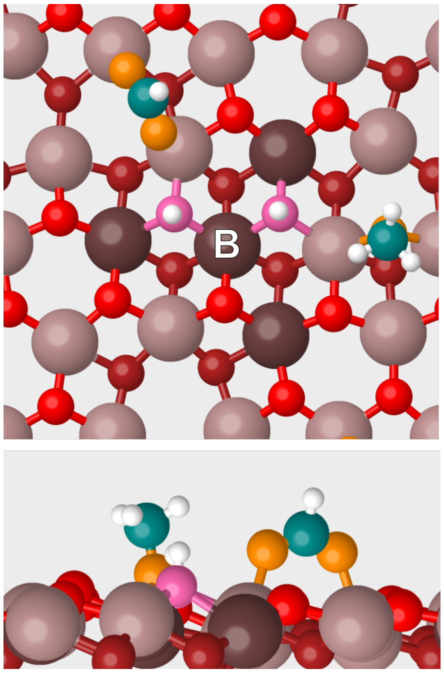

Figure S8: Formic acid and methanol adsorbed in the same $In_2O_3(111)$ unit cell for C 1s ΔCLS calcualtion.

PBE functional as a difference in the total energy of the system with the core hole either on the methanol or on the formic acid molecule. The calculated ΔCLS was 1.42 eV, clearly not in agreement with the experimental value of 2.6 eV. Previous calculations have shown that non-local exchange effects included in hybrid exchange-correlation functionals can lead to better agreement between the calculated CLS and experimental XPS data.[S3,S4] To investigate this effect for CLS of $In_2O_3(111)$ surface species, the C 1s ΔCLS of various carbon containing adsorbates were calculated using PBE and HSE06 functionals, accompanied by Bader charge analysis. The C 1s ΔCLS and Bader charges are presented in table S9. The HSE06 calculated C 1s ΔCLS for the formic acid is in much better agreement with the

Table S9: C 1s CLS (relative to methanol) and Bader charges (b.c.) on the carbon atom of carbon-containing adsorbates on stoichiometric $In_2O_3(111)$ calculated with PBE and HSE06.

	ΔCLS (eV)			b.c. on C, adsorbate			b.c. on C, MeOH		
Structure	PBE	HSE06	Δ	PBE	HSE06	Δ	PBE	HSE06	Δ
formic acid bridging	1.42	2.78	1.36	1.56	1.66	0.10	0.42	0.45	0.03
formic acid tilted	2.17	2.82	0.65	1.52	1.62	0.10	0.48	0.52	0.04
carbonate	2.42	2.92	0.50	2.05	2.22	0.17	0.46	0.49	0.03
formate ($HCOO+O_{ads}H$)	1.22	3.00	1.78	1.57	1.67	0.10	0.46	0.49	0.03
bicarbonate (HCO_3+O_sH)	3.27	3.91	0.64	2.08	2.22	0.14	0.47	0.49	0.02
bicarbonate ($HCO_3+O_{ads}H$)	3.82	4.65	0.83	2.18	2.29	0.11	0.43	0.46	0.03

experimental value. In general, all carbon containing adsorbates studied here present more positively shifted C 1s ΔCLS when calculated with HSE06 as compared to PBE. This is consistent with the improved charge separation achieved with the HSE06 functional, the effect of which can be seen as more positive Bader charges on the adsorbate carbon atoms.

References

(S1) Nelin, C. J.; Uhl, F.; Staemmler, V.; Bagus, P. S.; Fujimori, Y.; Sterrer, M.; Kuhlenbeck, H.; Freund, H.-J. Surface core-level binding energy shifts for MgO(100). *Phys. Chem. Chem. Phys.* **2014**, *16*, 21953–21956.

(S2) Bagus, P. S.; Nelin, C. J.; Levchenko, S. V.; Zhao, X.; Davis, E. M.; Kuhlenbeck, H.; Freund, H.-J. Surface core level BE shifts for CaO(100): insights into physical origins. *Phys. Chem. Chem. Phys.* **2019**, *21*, 25431–25438.

(S3) Van den Bossche, M.; Martin, N. M.; Gustafson, J.; Hakanoglu, C.; Weaver, J. F.; Lundgren, E.; Grönbeck, H. Effects of non-local exchange on core level shifts for gas-phase and adsorbed molecules. *J. Chem. Phys.* **2014**, *141*, 034706.

(S4) Delesma, F. A.; Van den Bossche, M.; Grönbeck, H.; Calaminici, P.; Köster, A. M.; Pettersson, L. G. M. A Chemical View on X-ray Photoelectron Spectroscopy: the ESCA Molecule and Surface-to-Bulk XPS Shifts. *ChemPhysChem* **2018**, *19*, 169–174.
